# Dynamic alteration of intrinsic properties of the cerebellar Purkinje cell during the motor memory consolidation

**DOI:** 10.1186/s13041-023-01043-9

**Published:** 2023-07-10

**Authors:** Dong Cheol Jang, Geehoon Chung, Sun Kwang Kim, Sang Jeong Kim

**Affiliations:** 1 Department of Physiology, Neuroscience Research Center, Wide River Institute of Immunology, Seoul National University College of Medicine, 103, Daehak-ro, Jongno-gu, Seoul, 03087 Republic of Korea; 2 Department of Brain and Cognitive Science, College of Natural Science, Seoul National University, 1, Gwanak-ro, Gwanak-gu, Seoul, 08826 Republic of Korea; 3 Department of Physiology, College of Korean Medicine, Kyung Hee University, 26 Kyungheedae-ro, Dongdaemun-gu, Seoul, 02447 Republic of Korea; 4 Department of East-West Medicine, Graduate School, Kyung Hee University, 26 Kyungheedae-ro, Dongdaemun-gu, Seoul, 02447 Republic of Korea

**Keywords:** Eye movement learning, Vestibular ocular reflex, Consolidation, Purkinje cell, Intrinsic plasticity

## Abstract

Intrinsic plasticity of the cerebellar Purkinje cell (PC) plays a critical role in motor memory consolidation. However, detailed changes in their intrinsic properties during memory consolidation are not well understood. Here, we report alterations in various properties involved in intrinsic excitability, such as the action potential (AP) threshold, AP width, afterhyperpolarization (AHP), and sag voltage, which are associated with the long-term depression of intrinsic excitability following the motor memory consolidation process. We analyzed data recorded from PCs before and 1, 4, and 24 h after cerebellum-dependent motor learning and found that these properties underwent dynamic changes during the consolidation process. We further analyzed data from PC-specific STIM1 knockout (STIM1^PKO^) mice, which show memory consolidation deficits, and derived intrinsic properties showing distinct change patterns compared with those of wild-type littermates. The levels of memory retention in the STIM1^PKO^ mice were significantly different compared to wild-type mice between 1 and 4 h after training, and AP width, fast- and medium-AHP, and sag voltage showed different change patterns during this period. Our results provide information regarding alterations in intrinsic properties during a particular period that are critical for memory consolidation.

**Supplementary Information:**

The online version contains supplementary material available at 10.1186/s13041-023-01043-9.

## Introduction

Intrinsic plasticity is a long-lasting modification of intrinsic excitability that has been reported in various brain regions [1–5]. Being accompanied by synaptic plasticity, intrinsic plasticity can be induced bidirectionally [4, 6–8]. Previous studies have focused on changes in firing frequency to evaluate the magnitude of intrinsic plasticity, as this is the most representative feature of changes in neuronal excitability [4, 7]. Hence, little is known about how intrinsic properties, except the firing frequency, change after intrinsic plasticity. Investigating intrinsic properties, such as action potential (AP) onset time, threshold, half-width, and other factors, is essential to gain a comprehensive understanding of the physiological role of intrinsic plasticity and its underlying mechanisms [7].

Memory consolidation stabilizes newly acquired information for long-term storage [9]. The cerebellum participates in various memory consolidation processes, including motor memory and fear conditioning memory [10–21]. Cerebellar memory consolidation processes involve the firing frequency of the cerebellar Purkinje cell (PC) [13–17], which is critically affected by changes in synaptic activity [10–12], protein synthesis [18], and intrinsic plasticity of PC [20]. In this study, we sought to identify the detailed changes involved in the intrinsic plasticity in the memory consolidation process. Although several studies have reported persistent alterations in intrinsic excitability during the consolidation period [22, 23], it remains unknown which changes in intrinsic properties accompany persistent alterations during this process.

To achieve this goal, we used data from our previous study, which used a transgenic mouse model of cerebellar memory deficits [20]. Previously, we reported that PC-specific deletion of stromal interaction molecule 1 (STIM1^PKO^) in mice resulted in memory consolidation deficits after eye movement learning [19, 20]. Interestingly, these mice showed a deficit in intrinsic plasticity [19, 20] in both directions without affecting synaptic plasticity [19]. Considering these unique characteristics, we investigated the changes in intrinsic properties related to memory consolidation. In a previous study, we trained mice using a training protocol and successfully induced both synaptic and intrinsic long-term depression in the wild-type. In the case of the STIM1^PKO^, synaptic but not intrinsic plasticity was induced as expected. We then collected various data related to changes in intrinsic excitability at 1, 4, and 24 h after training [20]. In the current study, we investigated the changes in intrinsic properties at each time point and inquired into the significant changes related to consolidation levels in wild-type littermates and STIM1^PKO^ mice. We found that PC intrinsic plasticity includes various changes in intrinsic properties that are continuously modulated during the memory consolidation process in normal mice. Although the intrinsic properties of STIM1^PKO^ changed after training, the alteration patterns differed from those of wild-type littermates. This suggests that different ion channels are involved in the changes in the intrinsic properties at each time point, and the resulting changes determine the level of memory retention.

## Methods

### Animal and behavior test

PC-specific STIM1 knockout mice line was generated by crossing PCP2-Cre line with a STIM1-floxed line as reported in our previous study [19]. For all experiments, 9 to 11-week-old male mice were used. All surgical and behavior testing procedures were similar to our previous paper [19]. Eye movement training was applied by giving associative visuo-vestibular stimulation. The visual and vestibular stimulations were simultaneously rotated out-of-phase with ± 5° amplitude. Gain, which indicates the amount of memory, was calculated as the ratio of the response amplitude to the stimulus amplitude. A custom-built LabView (National Instrument) tool was used to collect and analyze data.

### Slice preparation and whole-cell recording

All procedures for slice preparation and whole-cell recording were similar to our previous paper [20]. Before sacrificing mice for the slice, the mice experienced eye movement training and waited for a particular time (1, 4 and 24 h). 300 μm of coronal cerebellar slices, which include bilateral flocculus, were obtained by cutting with a vibratome slicer (Leica, VT1200). The brain was cut in ice-cold NMDG cutting solution containing the following (in mM): 93 NMDG, 93 HCl, 2.5 KCl, 1.2 NaH_2_PO_4_, 30 NaHCO_3_, 20 HEPES, 25 Glucose, 5 sodium ascorbate, 2 Thiourea, 3 Sodium pyruvate, 10 MgSO_4_·7H_2_O, 0.5 CaCl_2_·2H_2_O (pH 7.3). Slices were transferred to a recovery chamber containing NMDG-cutting solution kept at 32 ˚C for 10 min and then incubated at room temperature for 1 h in standard artificial cerebrospinal fluid containing the following (in mM): 125 NaCl, 2.5 KCl, 1 MgCl_2_, 2 CaCl_2_, 1.25 NaH_2_PO_4_, 26 NaHCO_3_, 10 glucose. All recordings from the flocculus were performed in the microzone where the floccular midline subregion is responsible for horizontal eye movement [24].

Brain slices were placed in a recording chamber on an Olympus microscope (BX50WI) stage and perfused with standard artificial cerebrospinal fluid. EPC9 amplifier with PatchMaster software (HEKA Elektronik) and a multiclamp 700B amplifier with pClamp 10 (Molecular Device) were used to amplify signals. Signals were collected with 20 kHz sampling frequency and filtered at 2 kHz. Inhibitory inputs were blocked by 100 μM picrotoxin (Sigma). Patch pipettes (3–4 MΩ) were pulled from borosilicate glass and filled with internal solution containing the following: 9 KCl, 10 KOH, 120 K-gluconate, 3.48 MgCl_2_, 10 HEPES, 4 NaCl, 4 Na_2_ATP, 0.4 Na_3_GTP and 17.5 sucrose (pH 7.25). In our recordings, membrane potentials were not corrected for the liquid junction potential, calculated 15.8 mV. The bridge balance was compensated after the whole cell was made. We discarded the data if the holding current when the cell was held at -70 mV was lower than -500 pA or series resistance was changed by more than 20%. The excitability of the cerebellar Purkinje cells was measured by injecting + 600 pA for 500 ms from -70 mV of baseline potential. The voltage sag and input resistance (R_in_) was measured by applying hyperpolarizing current injection from -100 pA to -500 pA with increments of -100 pA for 1 s. As an extension of our previous report on LTD-IE [4], all recordings were done at room temperature.

### Data analysis and statistics

The action potential (AP) onset time was measured by calculating the time delay from the acquisition of the current injection to the first spike. The AP threshold was determined by measuring the membrane potential where its velocity entered the range of 30–60 mV/ms [25]. AP amplitude was calculated as a difference between the threshold and positive peak. The full width at half maximum (FWHM) of AP was measured by calculating the time duration at the half maximum voltage of AP. The medium afterhyperpolarization (mAHP) was calculated as the voltage difference between the negative peak after the spike train and -70 mV baseline. The fast afterhyperpolarization (fAHP) was measured by subtracting the negative peak of AP from the AP threshold. To calculate the instantaneous frequency, we measured the interspike interval (ISI) by subtracting the peak time of each AP and the inverted ISI value. The amount of voltage sag determined as difference between the lowest peak and steady-state voltage during the hyperpolarizing current injection, and the R_in_ was calculated from the steady-state voltage and injected hyperpolarizing current. All data were imported and analyzed by custom-built python analysis code.

All statistical analyses were performed using Graphpad Prism 9. Two sample t-test, One-way ANOVA with Fisher’s LSD post-hoc test and the two-way ANOVA test were used. All graphs are shown as mean ± SEM. The asterisks *, **, and *** indicate p < 0.05, p < 0.01, and p < 0.001, respectively. Detailed statistical methods and n for each experiment are written in the figure legends.

## Results

### PC intrinsic plasticity involves changes in the rheobase current, AP onset time, threshold, and amplitude

**Fig. 1:**
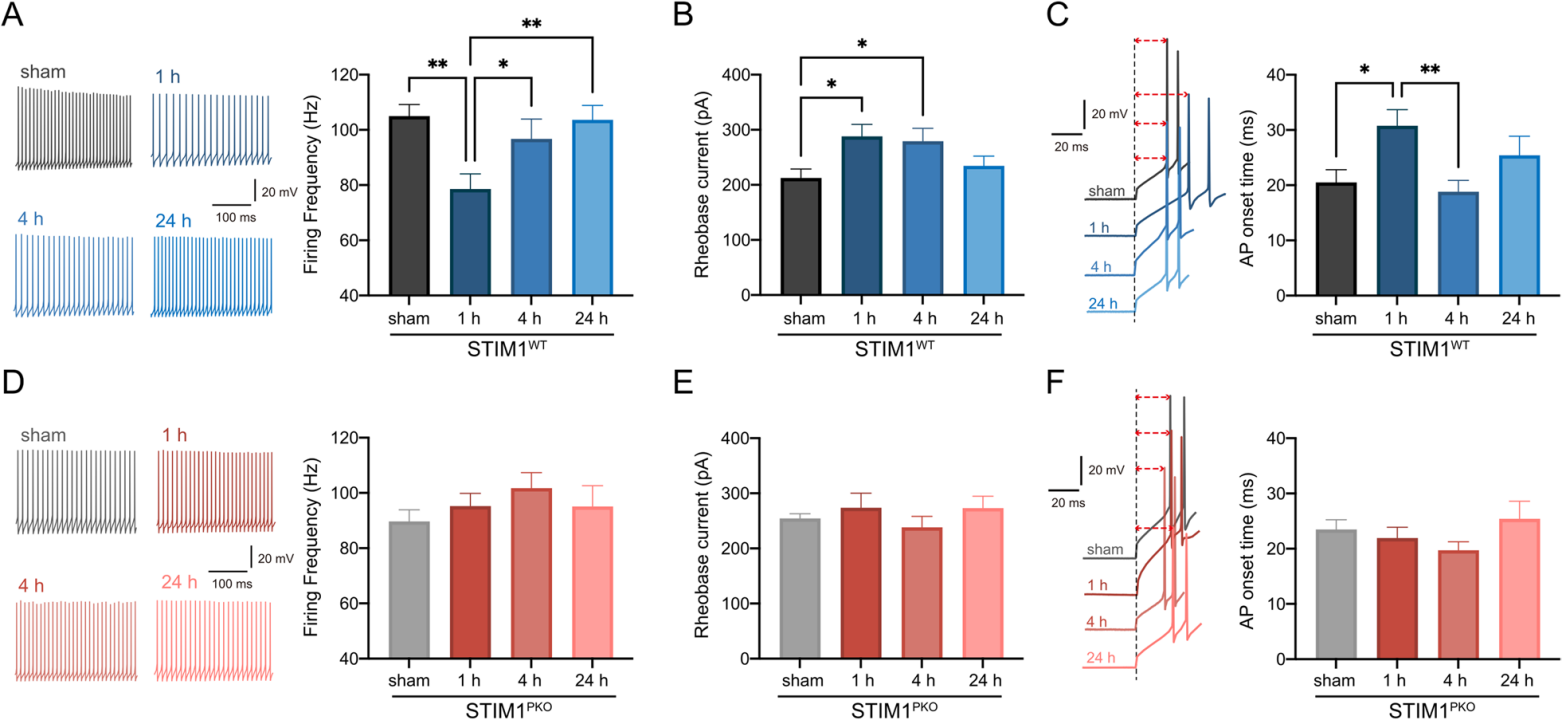
LTD-IE includes firing frequency, rheobase current and AP onset time. **A** Firing frequency of wild-type through time. Firing frequency at the 1 h time point was significantly lower than all other time points (F _3, 53_ = 3.64, p = 0.018; sham vs. 1 h, p = 0.004; 1 h vs. 4 h, p = 0.045; 1 h vs. 24 h, p = 0.005). **B** Rheobase current of wild-type through time. Rheobase currents at 1- and 4 h time points were significantly higher than sham control (F _3, 53_ = 3.13, p = 0.033; sham vs. 1 h, p = 0.017; sham vs. 4 h, p = 0.018). **C** AP onset time of wild-type through time. AP onset time was significantly altered at the 1 h time point (F _3, 53_ = 3.12, p = 0.033; sham vs. 1 h, p = 0.020; 1 h vs. 4 h, p = 0.008). **D** Firing frequency of STIM1^PKO^ through time. There were no significant differences in firing frequency (F _3, 53_ = 1.31, p = 0.280). **E** Rheobase current of STIM^PKO^ through time. The rheobase current was not significantly altered in STIM1^PKO^ (F _3, 53_ = 0.737, p = 0.535) **F** AP onset time of STIM1^PKO^ through the time. There were no significant alterations (F _3, 53_ = 1.46, p = 0.237). Sample numbers of wild-type (sham n = 15, 1 h n = 10, 4 h n = 15, 24 h n = 17) and STIM1^PKO^ (sham n = 16, 1 h n = 15, 4 h n = 16, 24 h n = 10) are the same for all panels. One-way ANOVA with post hoc Fisher’s LSD test was used for all panels. The graphs are shown as mean ± SEM. *p < 0.05, **p < 0.01

**Fig. 2:**
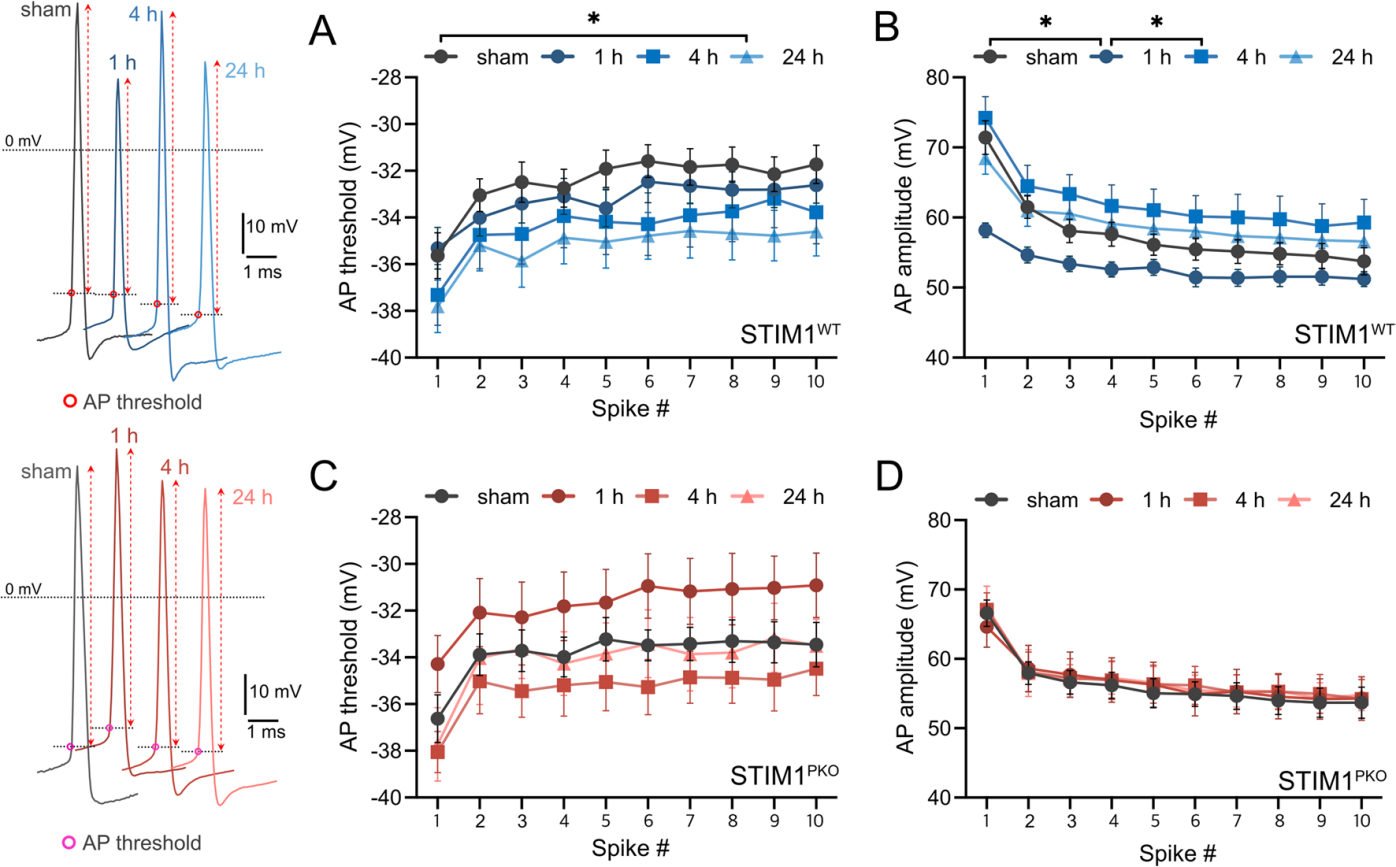
LTD-IE affects the AP threshold and amplitude. In representative AP traces, AP thresholds are pointed by red (wild-type) or magenta circle (STIM1^PKO^), and red arrows indicate AP amplitudes. **A** AP threshold of wild-type through time. AP threshold has been gradually decreased. It became significantly lower at the 24 h time point than the sham control (F _1, 30_ = 4.35, p = 0.046). **B** AP amplitude of wild-type through time. AP amplitude was significantly altered at the 1 h time point compared to sham control and the 4 h time point (sham vs. 1 h, F _1, 23_ = 5.63, p = 0.026; 1 h vs. 4 h, F _1, 23_ = 6.32, p = 0.019). **C**, **D** AP thresholds and amplitudes were not significantly altered between the time points. Sample numbers of wild-type (sham n = 15, 1 h n = 10, 4 h n = 15, 24 h n = 17) and STIM1^PKO^ (sham n = 16, 1 h n = 15, 4 h n = 16, 24 h n = 10) are the same for all panels. Two-way ANOVA test was used for all panels. The graphs are shown as mean ± SEM. *p < 0.05

We first investigated changes in the firing frequency of PCs over time following a training session for cerebellar learning. As we previously reported [20], the firing frequency was lowest in the group at the 1 h time point. Firing frequency recorded at 4 and 24 h after training showed a significantly increased firing frequency compared to that at 1 h, indicating that the firing frequency recovered gradually to a baseline level (sham) within 24 h (Fig. 1A). We then investigated the changes in the rheobase current, AP onset, and AP threshold, which are closely related to the firing frequency. Consistent with firing frequency changes, the rheobase current and AP onset time increased significantly at 1 h after training (Fig. 1B, sham vs. 1 h, p = 0.026; Fig. 1C, sham vs. 1 h, p = 0.020). The statistical significance of the AP onset time recovered by 4 h after training as the firing frequency (Fig. 1A, 1 h vs. 4 h, p = 0.045; Fig. 1E, 1 h vs. 4 h, p = 0.008), but the rheobase current remained significantly increased at this time point (Fig. 1C, sham vs. 4 h, p = 0.029). Remarkably, the AP threshold was not altered at the 1 and 4 h time points but was significantly reduced at the 24 h time point (Fig. 2A, sham vs. 24 h, p = 0.046, F_1, 30_ = 4.35). We interpreted that the AP threshold reduced continuously from the early stage and reached statistical significance at the end of consolidation, that is, the 24 h time point (Fig. 2A). These results indicate that although firing frequency at the 24 h time point was comparable to that of sham controls (i.e., baseline level), properties related to intrinsic excitability did not return to sham control levels. Instead, a gradual decrease in the AP threshold over 24 h implied that PC intrinsic plasticity occurred for at least 24 h following the end of the training session, with compensation for changes in the AP threshold. AP amplitude was considerably reduced after training (Fig. 2B). At the 1 h time point, AP amplitude was significantly reduced (Fig. 2B, sham vs. 1 h, p = 0.026, F_1, 23_ = 5.63) and reversed at 4 h after training (Fig. 2B, 1 h vs. 4 h, p = 0.019, F_1, 23_ = 6.32). In the case of STIM1^PKO^, there were no significant alterations in these properties (Fig. 2C, D), indicating that these alterations were not involved when the STIM1^PKO^ mice underwent training.

### PC intrinsic plasticity involves changes in AP width with up- and downstroke speeds

**Fig. 3:**
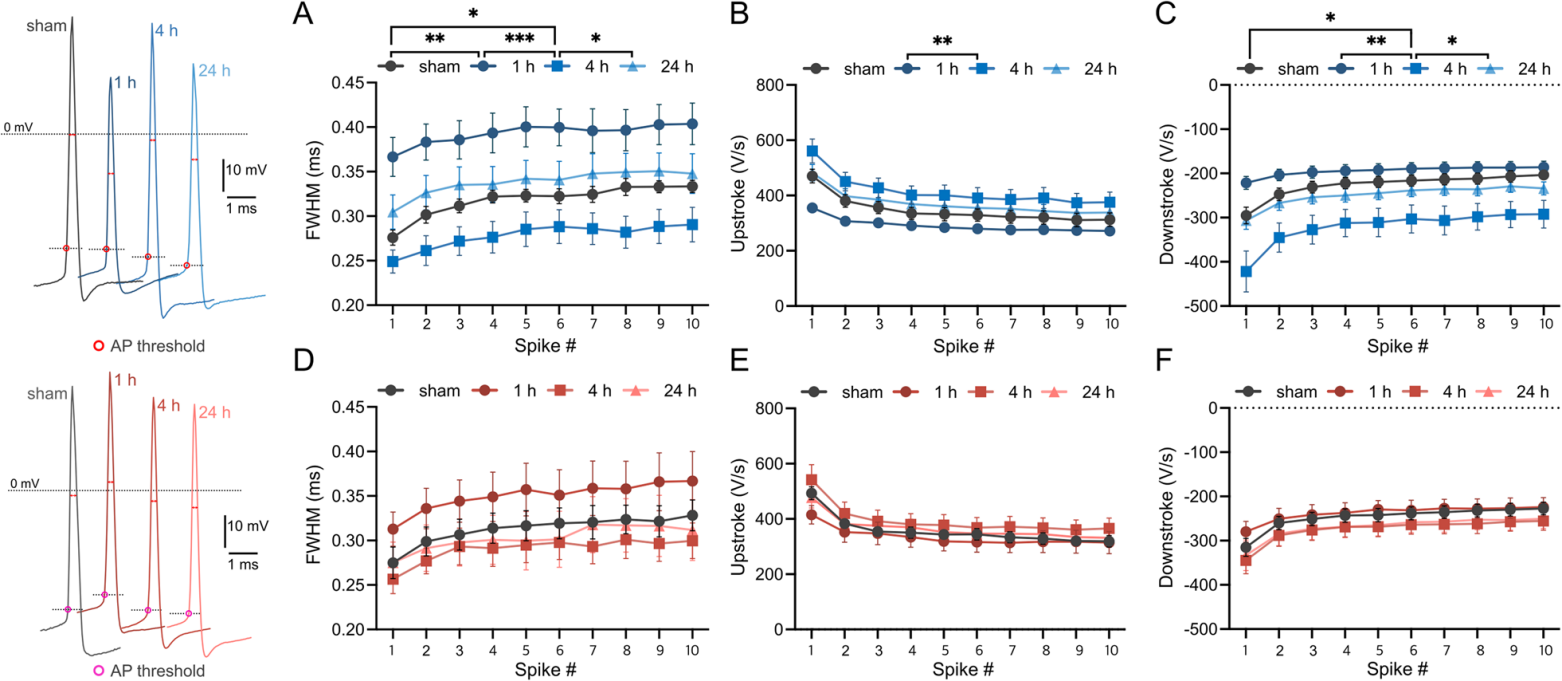
LTD-IE affects AP shape differently between wild-type and STIM1^PKO^. In representative AP traces, AP thresholds are pointed by red (wild-type) or magenta circle (STIM1^PKO^), and red arrows indicate FWHM. **A** FWHM of wild-type through the time. FWHM was significantly increased at 1 h after training compared to sham control (F _1, 23_ = 14.0, p = 0.001). The value of FWHM at the 4 h time point was significantly lower than all other time points (sham vs. 4 h, F _1, 28_ = 4.59, p = 0.041; 1 h vs. 4 h, F _1, 23_ = 17.3, p < 0.001; 4 h vs. 24 h, F _1, 30_ = 4.92, p = 0.034). **B** Upstroke speed of wild-type through time. Upstroke speed was significantly increased between 1 and 4 h time points (1 h vs. 4 h, F _1, 23_ = 8.49, p < 0.008) **C** Downstroke speed of wild-type through the time (sham vs. 4 h, F _1, 28_ = 5.82, p = 0.023; 1 h vs. 4 h, F _1, 23_ = 9.27, p = 0.006; 4 h vs. 24 h, F _1, 30_ = 4.36, p = 0.045). **D**–**F** STIM1^PKO^ showed no statistically significant alterations in FWHM, up- and down-stroke speeds. Sample numbers of wild-type (sham n = 15, 1 h n = 10, 4 h n = 15, 24 h n = 17) and STIM1^PKO^ (sham n = 16, 1 h n = 15, 4 h n = 16, 24 h n = 10) are the same for all panels. Two-way ANOVA was used for all panels. The graphs are shown as mean ± SEM. *p < 0.05, **p < 0.01, ***p < 0.001

Next, we analyzed changes in properties related to AP width, including full-width at half-maximum (FWHM), upstroke, and downstroke of the spikes. Rapid changes in FWHM were observed in wild-type mice (Fig. 3A), and these changes were based on the reduction in upstroke and downstroke speeds (Fig. 3B, C). The FWHM at the 1 h time point was significantly higher than that at baseline (sham vs. 1 h, p = 0.001, F_1, 23_ = 14.0). Although there were no statistically significant differences, both up- and downstroke speeds decreased at the 1 h time point (Fig. 3B, sham vs. 1 h, p = 0.117, F_1, 23_ = 2.64; Fig. 3C, sham vs. 1 h, p = 0.104, F_1, 23_ = 2.86). At the 4 h time point, however, the trend reversed, with FWHM being significantly lower than that at baseline (sham vs. 4 h, p = 0.041, F_1, 28_ = 4.59) and at the 1 h time point (1 h vs. 4 h, p < 0.001, F_1, 23_ = 17.3). At this time point, there were significant increases in the up- and downstroke speeds (Fig. 3B, 1 h vs. 4 h, p = 0.007, F_1, 23_ = 8.83; Fig. 3C, 1 h vs. 4 h, p = 0.004, F_1, 23_ = 10.6). The FWHM at the 24 h time point was higher than at the 4 h time point (4 h vs. 24 h, p = 0.034, F_1, 30_ = 4.92) and was not significantly different compared to the sham level. This alteration was based on the significant reduction in the downstroke speed at this time point (Fig. 3C, 4 h vs. 24 h, p = 0.046, F_1, 30_ = 4.32). In contrast, STIM1^PKO^ showed no alterations in these properties. FWHM was not significantly altered, although it slightly increased at the 1 h time point (Fig. 3D, sham vs. 1 h, p = 0.250, F_1, 29_ = 1.38). Both up- and downstroke speeds did not change after training (Fig. 3E, F).

### *PC intrinsic plasticity accompanies different patterns of afterhyperpolarization, spike frequency adaptation, and sag voltage in wild-type and STIM1*^*PKO*^* mice*

**Fig. 4:**
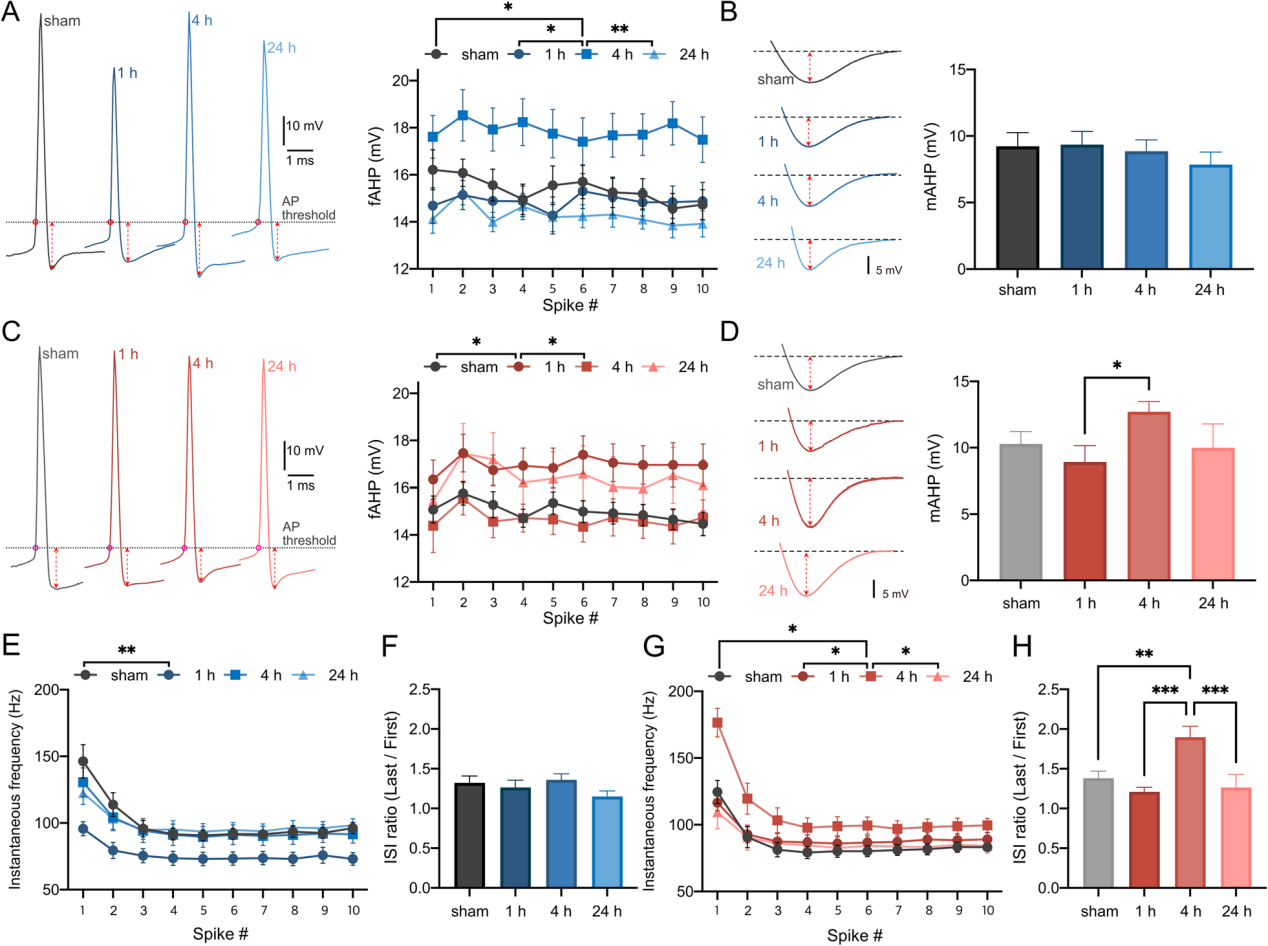
Afterhyperpolarization and spike frequency adaptation of both genotypes after LTD-IE induction. **A** fAHP of wild-type through time points. Representative AP traces are aligned to its AP thresholds (red circle) for comparison. The value of fAHP at the 4 h time point was significantly lower than all other time points (sham vs. 4 h, F _1, 28_ = 5.12, p = 0.032; 1 h vs. 4 h, F _1, 23_ = 5.72, p = 0.025; 4 h vs. 24 h, F _1, 30_ = 13.1, p = 0.001). **B** mAHP of wild-type through time points. There were no significant alterations between the time points (F _3, 53_ = 0.524, p = 0.667). **C** fAHP of STIM1^PKO^ through time points. Representative AP traces are aligned to its AP thresholds (magenta circles) for comparison. AP onset time was significantly altered at 1 h after training (sham vs. 1 h, F _1, 29_ = 5.68, p = 0.024; 1 h vs. 4 h, F _1, 29_ = 5.52, p = 0.026). **D** mAHP of STIM1^PKO^through time points. At the 4 h time point, mAHP was significantly increased than at the 1 h time point (p = 0.017). **E** The instantaneous frequency of wild-type through time. At the 1 h time point, the instantaneous frequency was significantly reduced compared to sham control (sham vs. 1 h, F _1, 23_ = 9.40, p = 0.005). **F** ISI ratio of wild-type through time. There were no changes throughout the period (F _3, 53_ = 1.45, p = 0.239). **G** The instantaneous frequency of STIM1^PKO^ through time. At the 4 h time point, the instantaneous frequency was significantly higher in comparison to all other groups (sham vs. 4 h, F _1, 30_ = 7.14, p = 0.012; 1 h vs. 4 h, F _1, 29_ = 4.32, p = 0.047; 4 h vs. 24 h, F _1, 24_ = 4.38, p = 0.047). **H** ISI ratio of STIM1^PKO^ through time. Similar to instantaneous frequency, the value at the 4 h time point was significantly higher than other time points (F _3, 53_ = 8.22, p < 0.001; sham vs. 4 h, p = 0.001; 1 h vs. 4 h, p < 0.001; 4 h vs. 24 h, p < 0.001). Sample numbers of wild-type (sham n = 15, 1 h n = 10, 4 h n = 15, 24 h n = 17) and STIM1^PKO^ (sham n = 16, 1 h n = 15, 4 h n = 16, 24 h n = 10) are the same for all panels. Two-way ANOVA was used for **A**, **C**, **E** and **G**. One-way ANOVA with posthoc Fisher’s LSD test was used for **B**, **D**, **F** and **H**. The graphs are shown as mean ± SEM. *p < 0.05, **p < 0.01, ***p < 0.001

**Fig. 5:**
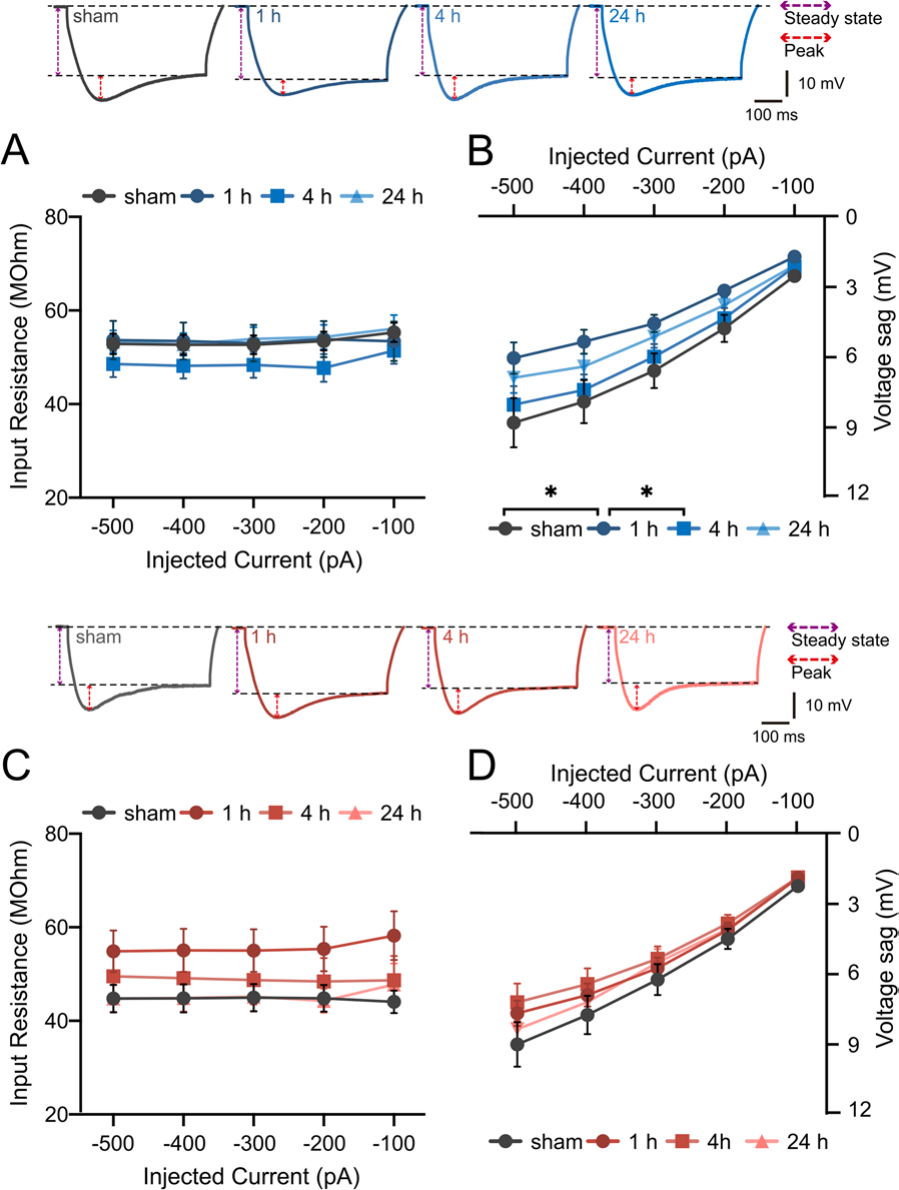
Sag voltage was altered after LTD-IE induction. **A** R_in_ was not altered in wild-type. **B** Sag voltage at the 1 h time point was significantly lower than the sham and the 4 h time point (sham vs. 1 h, F _1, 28_ = 4.50, p = 0.043; 1 h vs. 4 h, F _1, 25_ = 4.31, p = 0.048). **C** R_in_ was not significantly altered in STIM1^PKO^, although there was elevation at the 1 h time point (sham vs. 1 h, F _1, 26_ = 4.06, p = 0.054). (D) There were no significant changes in sag voltage through time. Sample numbers of wild-type (sham n = 18, 1 h n = 12, 4 h n = 15, 24 h n = 21) and STIM1.^PKO^ (sham n = 14, 1 h n = 14, 4 h n = 14, 24 h n = 9) are the same for all panels. Two-way ANOVA was used for all panels. The graphs are shown as mean ± SEM. *p < 0.05

We then analyzed the changes in fast- and medium-afterhyperpolarization (AHP) and spike frequency adaptation (SFA) following training. Similar to the rapid changes in FWHM in wild-type mice (Fig. 3A), there was a significant elevation of fAHP at the 4 h time point (Fig. 4A, sham vs. 4 h, p = 0.032, F_1, 28_ = 5.12; 1 h vs. 4 h, p = 0.025, F_1, 23_ = 5.72; 4 h vs. 24 h, p = 0.001, F_1, 30_ = 13.1). However, there was no alteration in mAHP levels in the wild-type (Fig. 4B, p= 0.675, F _3, 54_ = 0.513). In contrast, STIM1^PKO^ showed different patterns for these properties. There was a significant elevation of fAHP at the 1 h time point instead of the 4 h time point (Fig. 4C, sham vs. 1 h, p = 0.024, F_1, 29_ = 5.68), which was earlier than the wild-type. Interestingly, STIM1^PKO^ showed an increase in mAHP at the 4 h time point, which was not observed in wild-type littermates (Fig. 4D, 1 h vs. 4 h, p = 0.017). The instantaneous frequency of wild-type mice decreased significantly at the 1 h time point (Fig. 4E, sham vs. 1 h, p = 0.005, F_1, 23_ = 9.40) and returned to baseline levels at the 4 h time point. However, there was no change in the ISI ratio (Fig. 4F). Interestingly, STIM1^PKO^ showed a significant increase in instantaneous frequency and ISI ratio at the 4 h time point (Fig. 4G and H). The alterations at the 4 h time point were significant at all other time points (Fig. 4G, sham vs. 4 h, p = 0.012, F_1, 30_ = 7.13; 1 h vs. 4 h, p = 0.047, F_1, 29_ = 4.31; 4 h vs. 24 h, p = 0.047, F_1, 24_ = 4.37; Fig. 4H, sham vs. 4 h, p = 0.006; 1 h vs. 4 h, p < 0.001; 4 h vs. 24 h, p = 0.002). Next, we analyzed alterations in the R_in_ and sag voltage during negative current injection. In the wild-type, the R_in_ did not change at any time point (Fig. 5A). However, the sag voltage decreased significantly at the 1 h time point and was restored at the 4 h time point (Fig. 5B, sham vs. 1 h, p = 0.043, F_1, 28_ = 4.50; 1 h vs. 4 h, p = 0.048, F_1, 25_ = 4.31). STIM1^PKO^ showed no significant alterations in either the R_in_ or sag voltage (Fig. 5C, D). Although there was a notable increase in R_in_ at the 1 h time point, it was not statistically significant (Fig. 5C, sham vs. 1 h, p = 0.054, F_1, 26_ = 4.06).

### *PC intrinsic properties of STIM1*^*PKO*^* mice are comparable to wild-type*

While wild-type mice showed time-dependent changes in firing frequency, rheobase, and AP onset time following training, STIM1^PKO^ mice showed no significant alterations compared to the baseline state (Fig. 1D–F). These results may be attributed to the occlusion of long-term depression of intrinsic excitability (LTD-IE) in STIM1^PKO^ mice, which is indicated by significantly lower levels of baseline firing frequency compared to wild-type mice [19]. As seen in the comparison between wild-type sham and post-training data (Figs. 2 and 3), intrinsic properties were significantly altered at the 1 or 4 h time points. We questioned whether plasticity deficiency in STIM1^PKO^ mice was accompanied by baseline alterations in intrinsic properties. To investigate this, we compared data from sham groups of wild-type and STIM1^PKO^ mice to determine whether there were any differences (Additional file 1: Fig. S1, Additional file 2: Fig. S2, Additional file 3: Fig. S3, Additional file 4: Fig. S4). As we reported, the STIM1^PKO^ sham control showed a significantly lower firing frequency than the wild-type sham control (Figure S1A, p = 0.037). No significant differences were found in the properties, including rheobase current, AP onset time (Additional file 1: Fig. S1B, C), AP threshold, AP amplitude, FWHM, up- and downstroke speeds (Additional file 1: Fig. S2), fAHP, mAHP (Additional file 1: Fig. S3A, B), and sag voltage (Additional file 1: Fig. S4B). The instantaneous frequency was slightly altered, but this change was not statistically significant (Additional file 1: Fig. S3C, p = 0.065, F_1, 30_ = 3.67). The R_in_ decreased significantly for STIM1^PKO^ (Additional file 1: Fig. S4A, p = 0.016, F_1, 30_ = 6.55), which is comparable to our previous results [19].

## Discussion

Cerebellar learning includes two types of plasticity: synaptic and intrinsic [5]. In vestibular ocular reflex learning, synaptic LTD and LTD-IE are induced after gain-up training [20]. Deletion of STIM1 in Purkinje cells causes a deficit in intrinsic plasticity induction [19], and STIM1-deletion mice show severe memory deficiencies [19, 20]. These results imply that intrinsic plasticity directly contributes to memory consolidation. From this point of view, we compared the excitability of PCs between wild-type and STIM1^PKO^. Comparing memory retention levels after eye movement learning from STIM1^PKO^ with wild-type littermates, the differences became significant at the 4 h time point [20]. Because the retention levels of both groups were the same until 1 h after training, the time between 1 to 4 h after training, the intermediate stage, would be the critical period for the consolidation deficit in STIM1^PKO^ mice. Intrinsic plasticity is thought to play a critical role in the intermediate stage of successful consolidation because no differences were observed in synaptic plasticity except intrinsic excitability until this stage [20]. In this study, wild-type and STIM1^PKO^ mice showed different patterns of changes in intrinsic properties at this stage. While wild-type littermates showed an increase in AP up- and downstroke with a reduction in FWHM and an increase in fAHP and sag voltage, STIM1^PKO^ showed a decrease in fAHP, and an increase in mAHP and SFA (Fig. 5). These results imply that changes in each genotype involve different channels, such as Na^+^, Ca^2+^-activated K^+^ (K_ca_), and hyperpolarization-activated cyclic nucleotide-gated (HCN) channels. We discuss the possible role of each channel in memory consolidation.

Voltage-gated Na^+^ channels (Nav) contribute to AP amplitude and upstroke speed by generating Na^+^ influx. Partial blocking or reduced expression of voltage-gated Na^+^ channels causes a notable decrease in AP amplitude and upstroke speed [26, 27]. Among the various types of voltage-gated Na^+^ channels expressed in the cerebellum [28, 29], Nav1.6 (*Scn8a*) and Navβ4 (*Scn4b*) play a critical role in PC neuronal excitability by contributing to the resurgent Na^+^ current [30–32]. We observed a reduction in excitability with decreased AP amplitude and upstroke speed at the 1 h time point in wild-type mice, and these reduced properties were recovered during the intermediate stage (Figs. 1A, 2B, and 3B). These results imply that the resurgent Na^+^ current might be reduced until 1 h after training and were then restored during the intermediate stage. In contrast, STIM1^PKO^ mice showed no alterations in excitability, AP amplitude, or upstroke speed (Figs. 1D, 2D, and 3E). We previously reported the dysregulation of cytosolic Ca^2+^ concentration in STIM1^PKO^ that leads to elevated intracellular Ca^2+^ concentrations [19]. Given that Ca^2+^ concentration is critically related to the permeability of Nav1.6 via CaMKII activity [33], persistently elevated intracellular Ca^2+^ concentrations in STIM1^PKO^ mice may cause inflexible alterations in Nav1.6 permeability.

PCs express various K^+^ channels, including voltage-gated K^+^ and Ca^2+^-activated K^+^ channels (Kca) [29]. K^+^ channels contribute to the repolarization of the AP by generating a K^+^ efflux. Blocking A-type voltage-gated K^+^ channels increases the FWHM and decreases the downstroke speed in PCs [7], and D-type voltage-gated K^+^ channels contribute to the intrinsic plasticity of hippocampal CA3 neurons [34]. Notably, we observed a significant reduction in FWHM and an increase in downstroke in wild-type mice during the intermediate stage, and these alterations recovered at the 24 h time point (Fig. 3A, C). These results suggest that K^+^ conductance significantly increased during the intermediate stage and recovered by 24 h after training. In addition to the reduction of FWHM, we observed an increased fAHP in wild-type mice at the 4 h time point (Fig. 4A), suggesting increased permeability of large-conductance Kca (BK channel) during the intermediate stage [35, 36]. The mAHP was not altered during the entire process (Fig. 4B). In contrast, STIM1^PKO^ mice showed increased mAHP and SFA with no alteration in FWHM and downstroke speed at the 4 h time point (Figs. 3D, F, and 4D), implying increased permeability of small-conductance Kca (SK channel) during the intermediate stage [7, 37, 38]. The different activation of Kca observed in wild-type and STIM1^PKO^ mice could potentially be attributed to the distinct distribution of SK and BK channels, leading to the utilization of distinct calcium sources [39, 40]. The distinct changes in the properties related to different K^+^ channels suggest the critical involvement of PC firing fidelity in the memory consolidation process. With regard to memory consolidation in the cerebellum, the intermediate stage has been reported as a period for transferring newly acquired memory toward the deep cerebellar nucleus by plastic changes in PC firing [15, 18]. The alteration of BK channel permeability is involved in this process, as it is paranodally expressed in the axon, thereby contributing to modulation of the firing fidelity of PCs [41]. In contrast to the critical role of BK channel activity at the intermediate stage in the precise transfer of acquired information in the cerebellum, the deletion of SK channels in PCs did not lead crucial defects in eye movement learning and consolidation [42]. The deficits in memory consolidation in STIM1^PKO^ mice may stem from insufficient fidelity of PC firing originating from altered K^+^ channel permeability, although further studies are needed to clarify this.

The HCN channels are well-known contributors to hyperpolarization-activated current (Ih) channels [43] and are densely expressed in the cerebellum [44]. Previous studies have reported an essential role of this channel in the modulation of AP firing. For example, pharmacological blockade of HCN channels in GABAergic interneurons or GABAergic globus pallidus neurons reduces the firing frequency [45, 46]. HCN channels are known to affect neuronal excitability by modulating R_in_ [46, 47]. Our sag voltage results (Fig. 5B) indicate that HCN channel activity in wild-type mice was reduced within 1 h after training and was restored during the intermediate stage, which is similar to the time course of changes in firing frequency (Fig. 1A). Interestingly, there was no alteration in the R_in_ of the PCs (Fig. 5A), presumably because of compensation by other ion channels. We did not observe any alterations in sag voltage in STIM1^PKO^ mice (Fig. 5D). As in the case of other channels, these results support the critical involvement of the Ca^2+^ concentration. HCN channel activity is affected by protein kinase C (PKC) activation [43, 47]. Because LTD-IE is also dependent on PKC signaling [4], disruption of the PKC pathway due to abnormal intracellular Ca^2+^ concentrations could cause abnormal intrinsic plasticity and HCN channel activity, leading to deficits in memory consolidation.

**Fig. 6:**
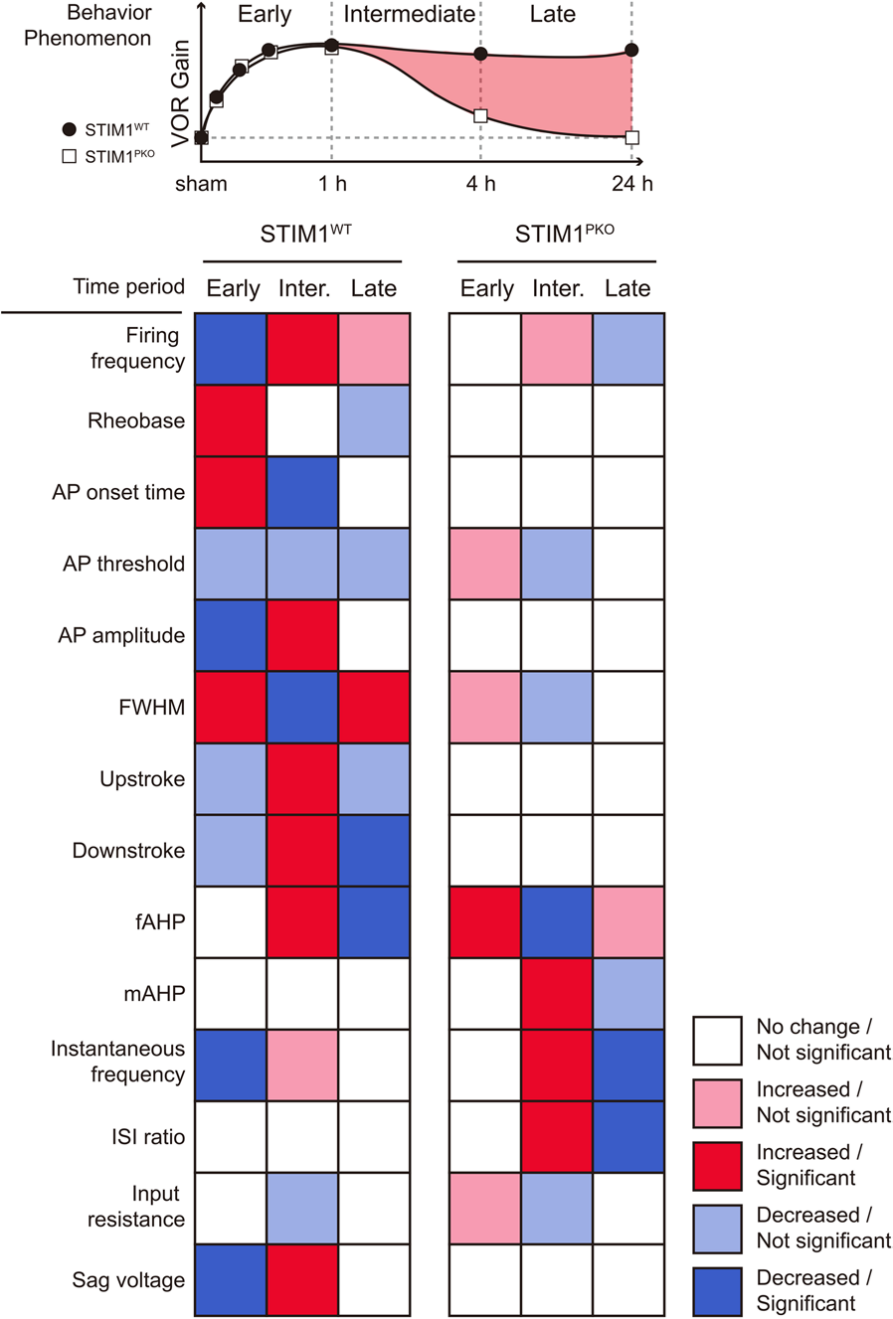
Schematic summary of the study. In behavior, wild-type and STIM1^PKO^ mice showed similar gain values in the early stage (0 h to 1 h). However, the gain of STIM1^PKO^ mice significantly decreased during the intermediate stage (1 h to 4 h) and decreased more during the late stage (4 h to 24 h) (upper). In the intrinsic properties, wild-type mice showed dynamic and significant alterations in most of the properties, except mAHP and ISI ratio. However, most properties were not altered in STIM1^PKO^ mice, and even it seems that altered properties in STIM1^PKO^ mice are opposite to wild-type mice (below)

This study demonstrated dynamic alterations in intrinsic properties during eye movement memory consolidation (Fig. 6). We compared the alterations between wild-type and consolidation-deficient mice and inferred the possible contribution of various ion channels to the consolidation process. This study had some limitations. First, our analysis was based solely on the memory consolidation of eye movement behavior, although the cerebellum is involved in the learning of other characteristic behaviors, such as eye blink conditioning or fear conditioning [48–50]. Several studies have reported excitability changes in PCs after eye blink conditioning or fear conditioning [50, 51]. Investigating the intrinsic properties of PCs in other types of cerebellum-related learning is expected to help elucidate the process of cerebellar memory consolidation. Secondly, our recordings were performed at room temperature to maintain the same recording conditions as in our previous study [4]. Because temperature is an influential factor for the opening probability of ion channels, the intrinsic properties may differ under physiological conditions. Finally, although STIM1^PKO^ showed a firm and clear phenotype, we only suggest a possible expectation in this study. Channel-specific modulation before, during, or after training is necessary to define the underlying mechanisms of the cerebellar memory process. Because these experiments are limited by several complications, computational modeling would be an acceptable approach.

## Supplementary Information

**Additional file 1: Fig S1.** Comparison of firing frequency, rheobase current and AP onset time between sham groups of wild-type and STIM1^PKO^. **A** STIM1^PKO^ showed reduced firing frequency than wild-type. **B**, **C** Rheobase currentand AP onset timesof both groups were not different. Sample numbers of wild-typeand STIM1^PKO^are the same for all panels. Two sample t-test was used for all panels. The graphs are shown as mean ± SEM. *p < 0.05.

**Additional file 2: Fig S2.** Comparison of AP shape with AP threshold between sham groups of wild-type and STIM1^PKO^. In representative AP traces, AP thresholds are pointed by redor magenta circle. There were no differences in **A** AP threshold, **B** AP amplitude, **C** FWHM**D** Upstrokeand **E** Downstroke. Sample numbers of wild-typeand STIM1^PKO^are the same for all panels. Two-way ANOVA was used for all panels. The graphs are shown as mean ± SEM.

**Additional file 3: Fig S3.** Comparison of afterhyperpolarization and spike frequency adaptation between sham groups of wild-type and STIM1^PKO^. There were no significant differences in **A** fAHP**B** mAHP. **C** Instantaneous frequency showed differences but could not fulfill statistical significance**D** ISI ratio of both groups were on the same level. Sample numbers of wild-typeand STIM1^PKO^are the same for all panels. Two-way ANOVA was used for **A** and **C**. Two sample t-test was used for **B** and **D**. The graphs are shown as mean ± SEM. *p < 0.05, **p < 0.01.

**Additional file 4: Fig S4.** Comparison of R_in_ and sag voltage between sham groups of wild-type and STIM1^PKO^. **A** The R_in_ of wild-type was significantly larger than STIM1^PKO^. **B** There were no significant differences in the sag voltage. Sample numbers of wild-typeand STIM1^PKO^are the same for all panels. Two-way ANOVA was used for all panels. The graphs are shown as mean ± SEM. *p < 0.05

## Data Availability

The datasets used and/or analyzed during the current study are available from the corresponding author upon reasonable request.

## Abbreviations

PC: Purkinje cell
STIM1: Stromal interaction molecule 1
STIM1^PKO^: Purkinje cell-specific STIM1 knockout
LTD-IE: Long-term depression of intrinsic excitability
LTP-IE: Long-term potentiation of intrinsic excitability
AP: Action potential
FWHM: Full-width half-maximum
fAHP: Fast afterhyperpolarization
mAHP: Medium afterhyperpolarization
SFA: Spike frequency adaptation
R_in_: Input resistance
Nav: Voltage-gated Na^+^ channels
ER: Endoplasmic reticulum
BK channel: Large-conductance Ca^2+^-activated K^+^ channel
SK channel: Small-conductance Ca^2+^-activated K^+^ channel
HCN channel: Hyperpolarization-activated cyclic nucleotide-gated channel
