# Supplementary figures and images for: Dynamic alteration of intrinsic properties of the cerebellar Purkinje cell during the motor memory consolidation
Source: Mol Brain. 2023 Jul 10;16:58. doi: 10.1186/s13041-023-01043-9 (PMC10334628; doi:10.1186/s13041-023-01043-9)

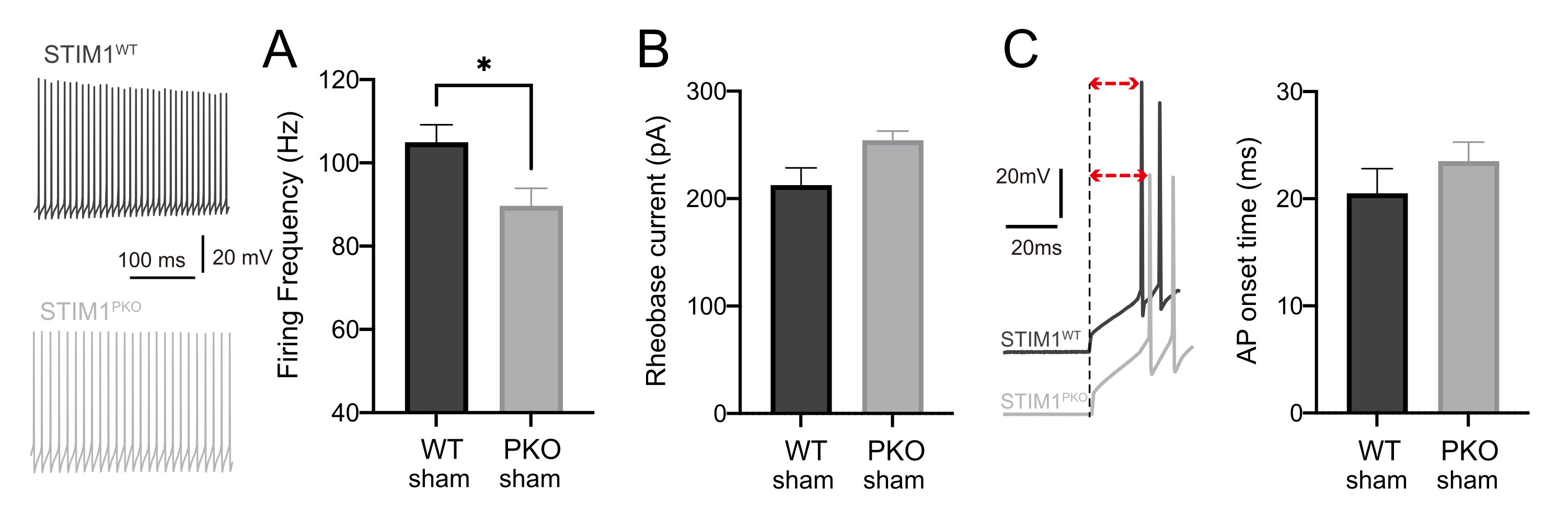

Supplement: Supplementary file 1 — Additional file 1: Fig S1. Comparison of firing frequency, rheobase current and AP onset time between sham groups of wild-type and STIM1PKO. A STIM1PKO showed reduced firing frequency than wild-type. B, C Rheobase currentand AP onset timesof both groups were not different. Sample numbers of wild-typeand STIM1PKOare the same for all panels. Two sample t-test was used for all panels. The graphs are shown as mean ± SEM. *p < 0.05. [file 13041_2023_1043_MOESM1_ESM.png]

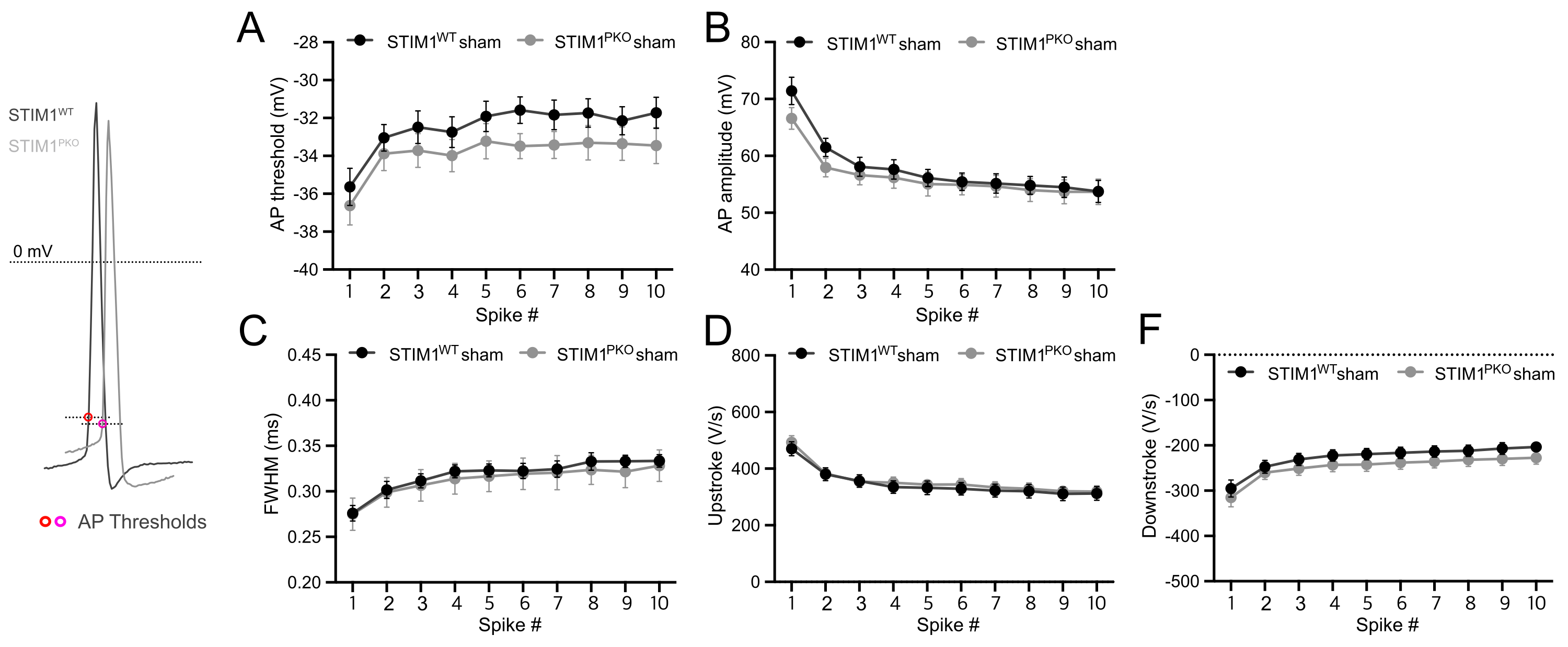

Supplement: Supplementary file 2 — Additional file 2: Fig S2. Comparison of AP shape with AP threshold between sham groups of wild-type and STIM1PKO. In representative AP traces, AP thresholds are pointed by redor magenta circle. There were no differences in A AP threshold, B AP amplitude, C FWHMD Upstrokeand E Downstroke. Sample numbers of wild-typeand STIM1PKOare the same for all panels. Two-way ANOVA was used for all panels. The graphs are shown as mean ± SEM. [file 13041_2023_1043_MOESM2_ESM.png]

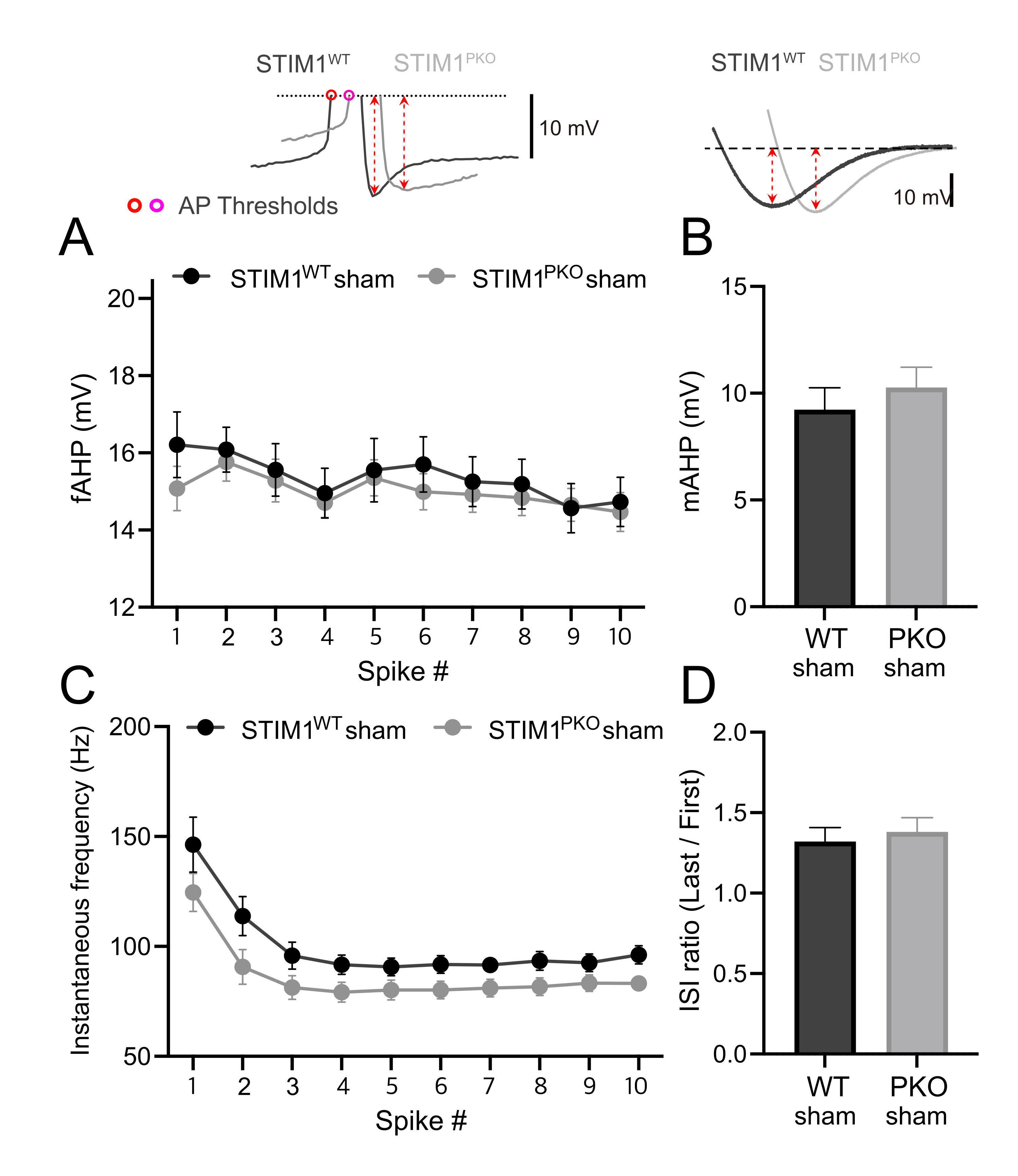

Supplement: Supplementary file 3 — Additional file 3: Fig S3. Comparison of afterhyperpolarization and spike frequency adaptation between sham groups of wild-type and STIM1PKO. There were no significant differences in A fAHPB mAHP. C Instantaneous frequency showed differences but could not fulfill statistical significanceD ISI ratio of both groups were on the same level. Sample numbers of wild-typeand STIM1PKOare the same for all panels. Two-way ANOVA was used for A and C. Two sample t-test was used for B and D. The graphs are shown as mean ± SEM. *p < 0.05, **p < 0.01. [file 13041_2023_1043_MOESM3_ESM.png]

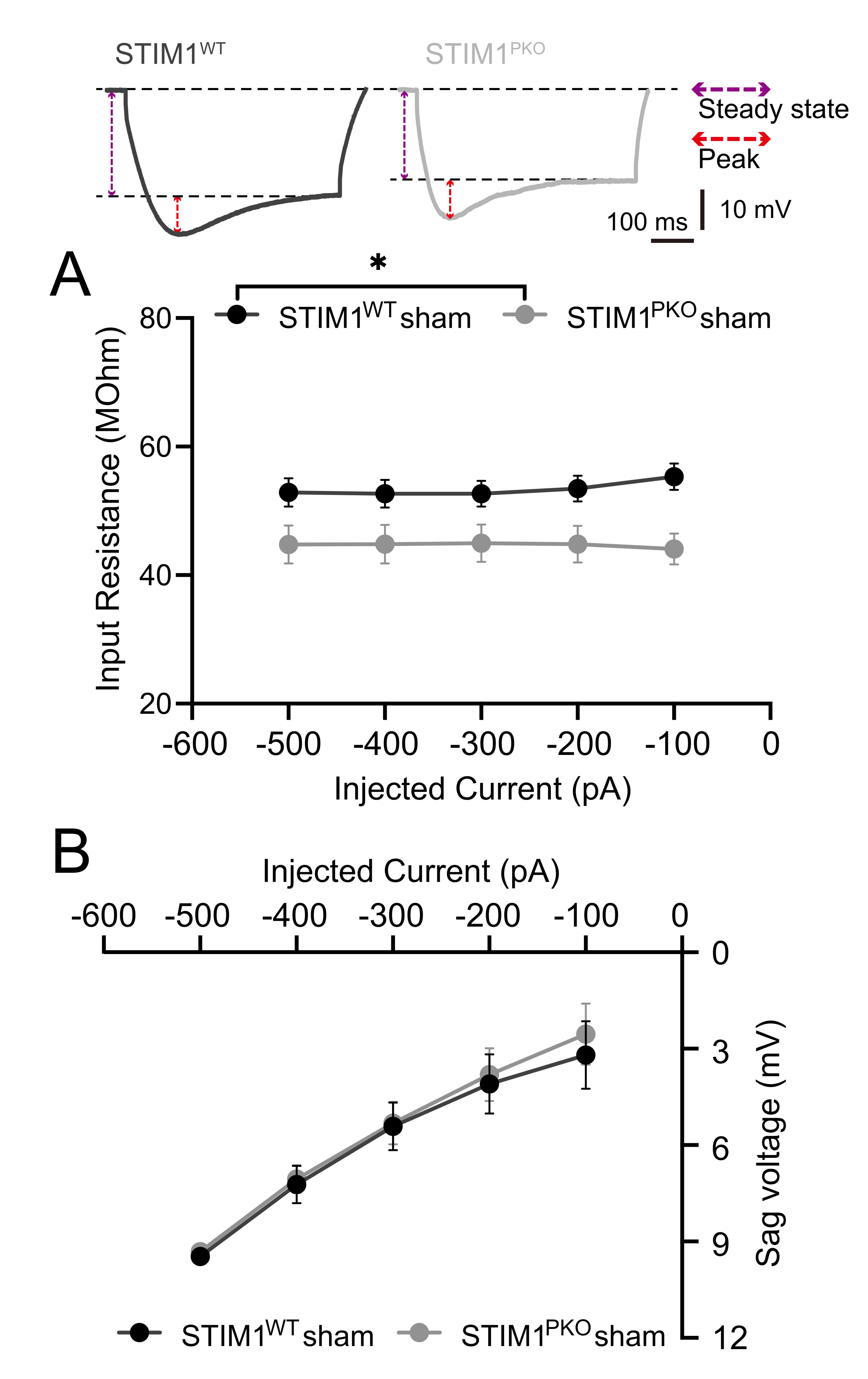

Supplement: Supplementary file 4 — Additional file 4: Fig S4. Comparison of Rin and sag voltage between sham groups of wild-type and STIM1PKO. A The Rin of wild-type was significantly larger than STIM1PKO. B There were no significant differences in the sag voltage. Sample numbers of wild-typeand STIM1PKOare the same for all panels. Two-way ANOVA was used for all panels. The graphs are shown as mean ± SEM. *p < 0.05 [file 13041_2023_1043_MOESM4_ESM.png]
